# Minimum Interference Channel Assignment Algorithm for Multicast in a Wireless Mesh Network

**DOI:** 10.3390/s16122056

**Published:** 2016-12-02

**Authors:** Sangil Choi, Jong Hyuk Park

**Affiliations:** 1 Department of Computer Science, Swaziland Christian University, P.O. Box A624 Swazi Plaza, Mbabane H101, Swaziland; sgilchoi@gmail.com; 2 Department of Computer Science & Engineering, Seoul National University of Science and Technology, Seoul 01811, Korea

**Keywords:** wireless mesh network, multicast communication, channel assignment, multi-radio multi-channel wireless mesh network

## Abstract

Wireless mesh networks (WMNs) have been considered as one of the key technologies for the configuration of wireless machines since they emerged. In a WMN, wireless routers provide multi-hop wireless connectivity between hosts in the network and also allow them to access the Internet via gateway devices. Wireless routers are typically equipped with multiple radios operating on different channels to increase network throughput. Multicast is a form of communication that delivers data from a source to a set of destinations simultaneously. It is used in a number of applications, such as distributed games, distance education, and video conferencing. In this study, we address a channel assignment problem for multicast in multi-radio multi-channel WMNs. In a multi-radio multi-channel WMN, two nearby nodes will interfere with each other and cause a throughput decrease when they transmit on the same channel. Thus, an important goal for multicast channel assignment is to reduce the interference among networked devices. We have developed a minimum interference channel assignment (MICA) algorithm for multicast that accurately models the interference relationship between pairs of multicast tree nodes using the concept of the interference factor and assigns channels to tree nodes to minimize interference within the multicast tree. Simulation results show that MICA achieves higher throughput and lower end-to-end packet delay compared with an existing channel assignment algorithm named multi-channel multicast (MCM). In addition, MICA achieves much lower throughput variation among the destination nodes than MCM.

## 1. Introduction

Wireless networks are a common communication environment these days. Wireless devices are widely spread out and we can use them every day. Especially, wireless mesh networks (WMNs) [1] are the most recently emerging technology that provides more reliability than mobile ad hoc networks (MANET). Typical WMNs usually consist of mesh clients, mesh routers, and gateways. The mesh clients are often laptops, cell phones, and other wireless devices, while the mesh routers forward data packets to and from the gateway nodes which usually connect to the Internet. WMNs can be implemented with various existing wireless technologies, including 802.11, 802.16, ZigBee, or cellular technologies.

Multicast communications can be useful for exchanging information between physical devices, such as wireless sensors. An individual physical device sends control or data packets to some control devices. These control nodes can collect and aggregate this information and send them to multiple interested devices or users. In addition, whenever the control machines receive packets, they distribute them to some destination devices or users. It is well known that multicast can transmit the same data flow to multiple destinations without producing the duplicated flow and wasting bandwidth.

One of the greatest challenges of applying multicast in machine-to-machine (M2M) communications [2,3,4] is how to deal with network interference between machines. Wireless mesh routers in WMNs are typically equipped with multiple network interfaces operating on different channels to increase network throughput. In a multi-radio multi-channel WMN, when two nearby nodes transmit on the same channel, they may interfere with each other and cause throughput decreases. Therefore, we need an efficient solution to reduce network interference to improve network throughput significantly. One of the best ways to decrease interference in WMNs is always to assign a different channel to each wireless device instead of the same channel. However, we do not have enough available channels to use at the same time. As a result, we need an efficient channel assignment mechanism to diminish the overall network interference in the network. In this work, we have proposed a minimum interference channel assignment (MICA) algorithm for multicast that accurately models the interference relationship between pairs of multicast tree nodes using the concept of the interference factor and assign channels to tree nodes to minimize interference within the multicast tree.

There are many studies on how to assign channels to nodes in WMNs [5,6,7,8,9,10,11,12,13,14]. All of these studies concentrate on unicast communications. On the other hand, channel assignment for multicast has been addressed recently [15,16,17,18,19,20,21,22,23]. In addition, there are emerging studies of resource allocation for small cell networks, called femtocell networks [24,25,26,27,28]. The femtocell network is a small coverage network to support home users. Femtocell networks suffer from cross-tier interference between macrocell and multiple femtocells as well. The channel assignment algorithm named multi-channel multicast (MCM) [17] suffers from low throughput caused by the hidden channel problem (HCP) [15]. Our proposed algorithm in this paper can get rid of HCP by considering every pair of nodes in the network. This algorithm allows nodes in a multicast tree to work with minimum interference. Our simulation results show that MICA achieves higher throughput and lower end-to-end packet delay compared with MCM. In addition, MICA accomplishes much lower throughput variation among the destination nodes than MCM.

This paper is organized as follows: Section 2 reviews the relevant literature regarding channel assignment for multicast in WMNs; we describe our proposed MICA algorithm and explain three procedures in detail in Section 3; Section 4 shows our simulation results, focusing on comparing the performance of MICA with that of MCM; and the last section finalizes the paper by providing conclusions.

## 2. Related Work

In this section, we review some of the related works in the area of channel assignment for multicast in multi-channel multi-radio WMNs. It is widely considered that there are two different types of channels in the study of channel assignment for multi-channel and multi-radio WMNs: orthogonal channels and partially-overlapped channels. In 802.11b/g network environments the use of 11 channels from channel number 1–11 is allowed [22]. Based on the IEEE 802.11b/g standard, if the channel gap is greater than, or equal, to 5 then two channels are non-overlapping. This means that three channel numbers 1, 6, and 11 are non-overlapping channels in 802.11b/g. Orthogonal channels can completely eliminate network interference between two nodes and dramatically increase network throughput. However, only three orthogonal channels are not enough to assign them to the network interfaces of wireless nodes to get rid of network interference completely. Thus, most of the studies of channel assignment consider partially-overlapping channels.

Previous studies on channel assignment problems in multi-channel multi-radio WMNs have been studied substantially for unicast communications [5,6,7,8,9,10,11,12,13,14]. However, recent studies on the channel assignment problem in WMNs focus on multicast manner [15,16,17,18,19,20,21,22,23]. Therefore, our work focuses on the channel assignment problem for multicast in WMNs.

**Figure 1:**
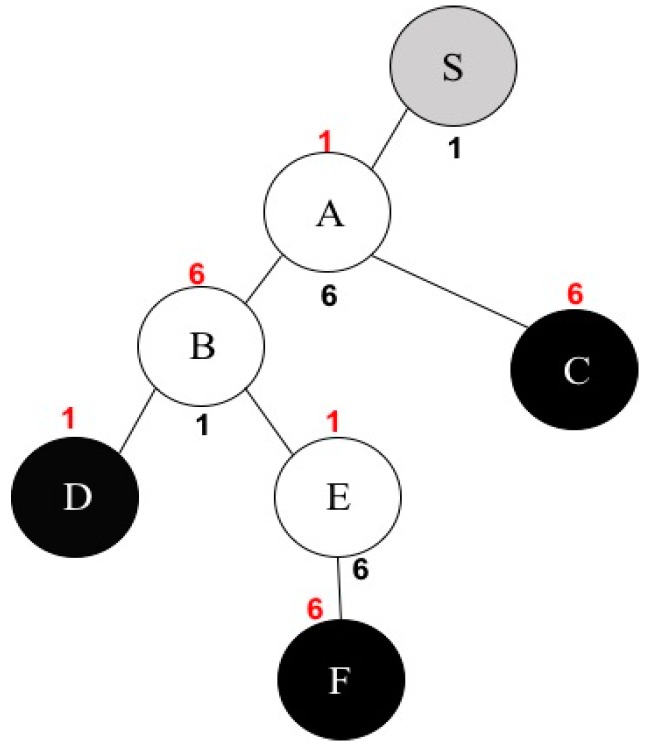
An example of channel assignment by MCM.

In [17], authors presented the MCM algorithm. The MCM algorithm is a channel assignment algorithm for multicast in multi-channel multi-radio wireless mesh network environments. This algorithm first constructs a multicast tree for multicast communications from a source to multi-receivers. In the multicast tree, there are three kinds of nodes: a source, relay nodes, and multi-receivers. A source node usually generates data packets, and a relay node receives packets from its relay node and forwards them to its children. Lastly, there are several receivers that just receive data from its parent node. In this algorithm, they assume each wireless node has two network interfaces: one for sending packets to its children and the other for receiving data from its relay node. Thus, each node needs two-channel information for these network interfaces. The main goal of MCM is to assign channel numbers to the sending and receiving interfaces, respectively, without network interference. The MCM algorithm uses an interference factor to estimate the level of interference between two nodes. The interference factor [17] is defined as the ratio of the interference range by the transmission range. The interference factor is also closely connected with channel separation [29] between two nodes. In the previous research, they conducted real experiments to measure the interference factor between two wireless peer-to-peer links. Table 1 shows the interference factors in an IEEE 802.11b network. As seen from this table, we can know that the interference range decreases as the channel separation increases. For instance, if the channel separation is greater than, or equal to, 5 then the interference factor value is zero, which means there is no interference between the two nodes. An example of channel assignment by MCM is shown in Figure 1. In this example, S is a source node, C, D, and F are multi-receivers, and A, B, and E are relay nodes. The black-colored numbers represent the channel number for each node’s sending interface and the red-colored number represents the channel number for each node’s receiving interface. The MCM channel assignment algorithm considers only one-hop neighboring nodes to decide the influence of interference. This approach may yield the HCP. The HCP takes place when two different nodes which are away from each other with a two-hop distance use the same channel, thus these two nodes can interfere with themselves. For example, node A receives packets from node S on channel 1, which means node A is located in the source node’s transmission range. Additionally, node A is within node B’s transmission range in Figure 1. Unfortunately, node B uses the same channel as the source node’s sending interface. If nodes S and B transmit their packets at the same time, there will be a collision at node A. This situation occurs because node B examines only the channel assigned to its one-hop neighbor A and does not consider the one assigned to two-hop neighbor S. Our new channel assignment algorithm thinks about every pair of nodes within the given network in order to minimize network interference and improve overall network throughput.

Nguyen et al. [15] provided a channel assignment algorithm named minimum interference multi-channel multi-radio multicast (M4). M4 allows nodes to broadcast the channel information to their neighbors whenever every node receives the broadcast message, it adds its own channel information and re-broadcasts the updated message to its neighbors. The M4 algorithm has definitely improved performance compared to MCM. It considers not only one-hop neighbors but also two-hop ones for channel assignment to solve HCP. However, if the physical distance of one node and its two-hop neighbors is close, there may be network interference between the two nodes. Therefore, M4 still has a network interference problem. Due to this reason, our proposed algorithm investigates every pair of nodes in the multicast tree to minimize network interference.

Jahanshahi et al. [18] proposed a layered binary integer programming (layered BIP)-based solution for the channel assignment in WMNs. The primary goal of layered BIP is to minimize the total number of links in the multicast tree and network interference. One drawback of the study is that there is no large difference to deal with multicast communication compared to unicast communication. In [19], authors also adopted an integer linear programming in order to minimize the carried load on the most-heavily loaded channel and maximize the residual capacity of the most heavily loaded node. They proposed a heuristic multicast tree construction and channel assignment algorithm called largest coverage and shortest path first (LC-SPF).

MCM and M4 mainly concentrate on minimizing network interference between nodes in order to increase network throughput and mitigate end-to-end packet delay. However, Yang et al. [20] proposed the load-based MCM (LMCM). This scheme selects a relay node based on the largest load value when it constructs a multicast tree. In this study, load means the number of subscribers served by the sub-tree rooted at the node. In addition, two channel assignment mechanisms are also proposed in the paper-load-based depth first search and load-based breadth first search.

Karimi et al. [21] proposed a heuristic channel assignment algorithm named progressive channel assignment (progressive CA). In progressive CA, channel assignment is decided according to a certain acceptable threshold value. The algorithm visits each node in the multicast tree using a breadth-first search to find the highest interference factor below the threshold. However, there is no mention about how to decide the threshold in the paper.

## 3. Minimum Interference Channel Assignment

The ultimate goal of the minimum interference channel assignment (MICA) algorithm is to assign communication channels to wireless devices properly for the purpose of minimizing network interference among nodes. Therefore, MICA achieves maximum throughput and minimum end-to-end packet delay for multicast in wireless mesh networks. In this section, we describe how the MICA algorithm works.

The MICA algorithm accepts a multicast tree as an input and produces a channel assignment for communicating interfaces of each wireless node in the tree. There are three steps in MICA. The first step is to calculate channel separation of all pairs of nodes in the network in order to avoid network interference. The second phase is to find whether there is a channel separation of zero (*CS_zero_*) among wireless nodes. This is why we could assign the same channel to those pairs of nodes as many times as possible. Lastly, MICA assigns an optimal channel numbers to all of the nodes in the network.

### 3.1. Calculating a Channel Separation of All Pairs of Nodes

MICA first computes the channel separation of all pairs of nodes in a multicast tree. In the first step, the algorithm considers only a pair of nodes which are not a leaf node in the tree. *Channel Separation* is defined as the difference in channel numbers which are used by the two pairs of nodes [29]. When calculating the channel separation of two pairs, MICA examines every pair of nodes in the tree to minimize network interference between two nodes. Algorithm 1 shows the algorithm of calculating channel separation of all pairs of nodes. This algorithm needs to calculate the channel separation of all pairs of nodes in the network, so the time complexity of the algorithm is Ο(n), where *n* is the total number of nodes in the network.
**Algorithm 1:** Calculating Channel Separation  1: **input**: *T*, a multicast tree  2: **output**: *CS<u, v>*, the channel separation of all pairs of nodes in *T*  3: **procedure** CALCULATING THE CHANNEL SEPARATION OF ALL PAIRS OF NODES  4: **for** each pair of nodes *(u, v)*
∈T do  5:  *C_u_* ← the set of *u*’s children  6:  *C_v_* ← the set of *v*’s children  7:  *MAX_u_* ← 0  8:  *MAX_v_* ← 0  9:  **for** each node *i_u_*
∈
*C_u_*
**do** 10:   *CS<v, i_u_>* ← channel separation between *v* and *i_u_* 11:   *MAX_v_* ← a maximum value between *MAX_v_* and *CS<v, i_u_>* 12:  **end** 13:  **for** each node *i_v_*
∈
*C_v_*
**do** 14:   *CS<u, i_v_>* ← channel separation between *u* and *i_v_* 15:   *MAX_u_* ← a maximum value between *MAX_u_* and *CS<u, i_v_>* 16:  **end** 17:  *CS<u, v>* ← a maximum value between *MAX_u_* and *MAX_v_* 18: **end**

The purpose of the first step is to compute the channel separation of all pairs of nodes in the multicast tree. First, MICA considers all pairs of nodes (*u*, *v*) in the given tree. Next, it attempts to obtain the channel separation between *u* and *v*’s children and *v* and *u*’s children.

We use an interference factor to compute the channel separation of two nodes. As mentioned before, the interference factor is defined as the ratio of an interference range to a transmission range. From this definition, we can obtain the interference range by multiplying the interference factor by the transmission range. If a physical distance of two comparing nodes is within the obtained interference range, these two nodes may interfere with each other. However, we can eliminate network interference between two nodes by increasing channel separation. According to Table 1, as the value of channel separation increases, an interference factor decreases. For example, if channel separation is greater than, or equal to, 5 there is no interference between two nodes in an 802.11b network. Consequently, we can get channel separation with no interference between two nodes if we can acquire channel separation in which the physical distance of two nodes is greater than the interference range of these two nodes. Finally, we can decide the final channel separation between *u* and *v* by choosing the maximum channel separation value among *u* and *v*’s children and *v* and *u*’s children.

**Figure 2:**
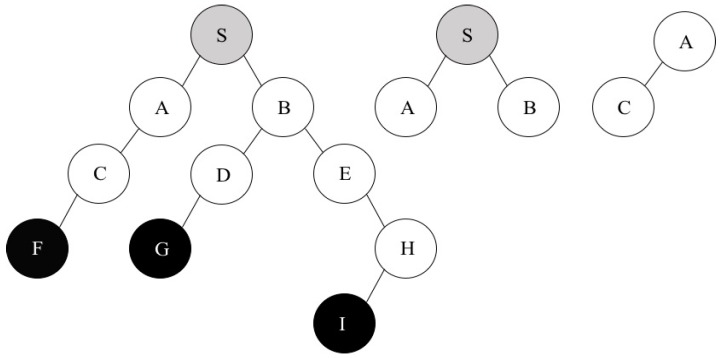
Multicast tree and children of S and A.

We assume that all wireless nodes have the same transmission range 250 m in the multicast tree shown in Figure 2. As mentioned before, we do not consider leaf nodes in the first step. Therefore, we only think about the combination of all nodes except F, G, and I in the tree. For instance, we want to calculate the channel separation of S and A. MICA always looks at the opponent’s children to totally eliminate network interference between two nodes. In the case of S and A pair, S’s children are A and B and A’s child is C. As a result, (S, C) and (A, B) pairs will be considered to calculate the channel separation of S and A.

Let us assume that the physical distance of S and C is 350 m and that of A and B is 420 m. If the channel separation of two arbitrary nodes is 1 then an interference factor is 1.6 at 2 Mbps of network bandwidth based on Table 1. Now, we can compute the interference range by multiplying 1.6 by 250 m (a given transmission range). Hence, the interference range between any nodes is 400 m in this case. This result means that if the distance of two nodes is within this amount of value then they might interfere with each other. Therefore, we should increase the channel separation of the two nodes in order to avoid network interference. Given by the scenario, the channel separation of S and C should be two and that of A and C is one to remove network interference. Finally, MICA can decide the maximum value (=2) as a result of the channel separation of S and A. This procedure can be applied to calculate the channel separation of all pairs of nodes except F, G, and I in the multicast tree shown in Figure 2.

### 3.2. Finding Channel Separation of Zero

Finding channel separation of zero is the second step. There are two situations in this step. The first scenario is that there is *CS_zero_* between two nodes. The other case is that there exists no pair of nodes with *CS_zero_*. In this step, we first find *CS_zero_* in a multicast tree. If there is no *CS_zero_*, we try to discover channel separation of a maximum value. The algorithm of finding *CS_zero_* is represented in Algorithm 2. Like the algorithm of calculating the channel separation in Section 3.1, the time complexity of finding channel separation of zero is also Ο(n), where *n* is the total number of nodes in the network.

*CS_zero_* means that there is no channel difference between two nodes. In other words, although two nodes use the same channel number at the same time, there is no interference between these two nodes. It is very important to find *CS_zero_* because available channel numbers are limited.

The second step takes a multicast tree and the channel separation obtained from the first step as the input. We define two sets of nodes, *S_D_* and *S_N_* in this phase. While *S_D_* contains nodes that acquire their own channel number, *S_N_* holds nodes that do not have a channel number. These sets are the output of the second step. For each pair of nodes, *u* and *v*, in a multicast tree, MICA investigates whether there is *CS_zero_* or not. If it exists, the given algorithm (Algorithm 2) assigns a specific channel number, a channel number 6, to the sending interface of *u* and *v*. Especially, we use a certain channel number 6 in this algorithm because this number is one of the orthogonal numbers as we mentioned before. Furthermore, it could be possible to eliminate network interference the first time as we discussed before. Then, *u* and *v* are allocated to *S_D_* and the rest of the nodes except *u* and *v* are assigned to *S_N_*. Next, if *S_D_* is not empty, the algorithm keeps searching if there is *CS_zero_* between node *i* in *S_N_* and all of the nodes in *S_D_*. Whenever obtaining *CS_zero_*, we can remove *i* from *S_N_* and put it into *S_D_*. This procedure continues until there is no *CS_zero_*. Otherwise, for every (*u*, *v*), we just select a channel separation of a maximum value between *u* and *v*. After that, we need to assign channel number 6 to one of these nodes and the summation of channel number 6 and the maximum channel separation value to the other node as its channel number. Finally, we put *u* and *v* to *S_D_* and all nodes except *u* and *v* to *S_N_*.
**Algorithm 2:** Finding Channel Separation of Zero  1: **input**: *T*, a multicast tree  2:    *CS<u, v>*, the channel separation of all pairs of nodes in *T*  3: **output**: *S_D_*, the set of nodes with a channel number for their sending interface  4:    *S_N_*, the set of nodes without a channel number for their sending interface  5: **procedure** FINDING CHANNEL SEPARATION OF ZERO  6: **for** each pair of nodes *(u, v)*
∈T do  7:  **if**
*CS<u, v>* == 0 **then**  8:   sending interface of *u* ← channel number 6  9:   sending interface of v ← channel number 6 10:   *S_D_* ← *u, v* 11:   *S_N_* ← all nodes except *(u, v)* in *T* 12:   **break** 13:  **end** 14: **end** 15: **if**
*S_D_*
≠∅
**then** 16:  **for** each node *i*
∈
*S_N_*
**do** 17:   **if**
*CS<i, j> == 0* for all *j*
∈
*S_D_*
**then** 18:    sending interface of *i* ← channel number 6 19:    remove *i* from *S_N_* 20:    *S_D_* ← *i* 21:   **end** 22:  **end** 23: **else** 24:  find *u*, *v*
∈
*T* such that *CS<u, v>* is a maximum 25:  sending interface of *u* ← channel number 6 26:  sending interface of *v* ← channel number 6 + *CS<u, v>* 27:  *S_D_* ← *u*, *v* 28:  *S_N_* ← all nodes except except *(u, v)* in *T* 29: **end**

Let us think about the following example to illustrate the second step. We assume that there exists *CS_zero_* between nodes A and D in the multicast tree shown in Figure 2, which means that the same channel number can be assigned to A and D. In this case, MICA allocates channel number 6 to these two nodes for their sending interface and put A and D to *S_D_*. After that, it keeps searching for a pair of nodes with *CS_zero_*. Nodes A and D have already been put in *S_D_*, so MICA needs to keep comparing all other nodes which are located in *S_N_* with the two nodes A and D, for instance, after checking both channel separation between A and H and channel difference between D and H. If these two channel separation values (*CS <*A, H*>* and *CS <*D, H*>*) are all zero, MICA can assign the same channel number as that of A and D to H and put it into *S_D_*. As a result, *S_D_* = {A, D, H} and *S_N_* = {S, B, C, E}. This process continues until all of the nodes in *S_N_* are examined.

There is one more situation in the second step. Let us assume that there is no pair of nodes with *CS_zero_* in the first place. In this scenario, MICA just chooses two nodes with a channel separation of a maximum value. Suppose that a channel separation of *A* and *H* has a maximum value of 5. Now, we can assign channel number 6 to A and 11 to H and put these two nodes into *S_D_* based on the given algorithm. Finally, all remaining nodes—S, B, C, D, and E—are located in *S_N_*. Therefore, *S_D_* = {A, H} and *S_N_* = {S, B, C, D, E}.

### 3.3. Assigning a Channel Number to All Nodes

In the final round, MICA needs to assign a channel number to all of the nodes in *S_N_*. The final process is well illustrated in Algorithm 3.
**Algorithm 3:** Assigning a Channel Number to All Nodes1: **input**: *T*, a multicast tree 2:    *S_D_*, the set of nodes with a channel number for their sending interface 3:    *S_N_*, the set of nodes without a channel number for their sending interface 4: **output**: *Channel assignment for all network interfaces* 5: *CS<u, v>*: the channel separation of all pairs of nodes in *T* 6: *CH_i_*: a channel number for node *i* 7: *COND_j_*: the set of conditions for deciding a channel number of node *j* 8: **procedure** ASSIGNING A CHANNEL NUMBER 9: **while**
*S_N_*
≠∅
do 10:  find *x*
∈
*S_N_*, *y*
∈
*S_D_* such that *CS<x, y>* is a maximum 11:  **for** each node *k*
∈
*S_D_*
**do** 12:   *COND_x_* ← [*CH_x_* >= *CH_k_* + *CS<x, k>* or *CH_x_* <= *CH_k_* – *CS<x, k>*] 13:   eliminate all invalid channel numbers 14:   $C{\tilde{OND}}_{x}$ ← *COND_x_*
− conflict conditions 15:    select *CH_x_* that satisfies all conditions ∈
$C{\tilde{OND}}_{x}$ 16:  **end** 17:  *x* ← *CH_x_* 18:  remove *x* from *S_N_* 19:  *S_D_* ← *x* 20: **end**

MICA takes *S_D_* and *S_N_* as inputs and produces a final channel assignment for all network interfaces of every node in a multicast tree. First, MICA compares a channel separation of each node *i* in *S_N_* with all nodes in *S_D_*. After finishing all comparisons, it selects one node which has a channel separation of a maximum value. Next, for each node *k* in *S_D_*, it can be possible for MICA to generate several conditions for the channel number of node *i*. One possible channel number of *i* might be greater than, or equal to, the summation of *CS < k*, *i >* and the channel number of *k*. The other could be less than, or equal to, the difference between *CS < k*, *i >* and the channel number of *k.* Based on the number of elements of *S_D_*, the number of conditions will be decided. After producing all possible conditions, MICA can choose a channel number for node *i*. Eventually, the selected channel number will be used for the sending interface of *i*. Now MICA can remove *i* from *S_N_* and put it into *S_D_*. This process keeps running until there are no elements in *S_N_*, which means the sending interface of all nodes in a multicast tree has its own channel number. Lastly, we can easily assign a channel number to each receiving interface of all nodes because the receiving interface of each node should be the same channel number as its parent’s sending interface to communicate with each other. The algorithm of assigning channel numbers is affected by the algorithm shown in Algorithm 2. Therefore, this channel assignment algorithm takes Ο(n) in the worst case. As a result, the overall time complexity of the proposed channel assignment algorithm takes Ο(n), where *n* is the total number of nodes in the network.

**Figure 3:**
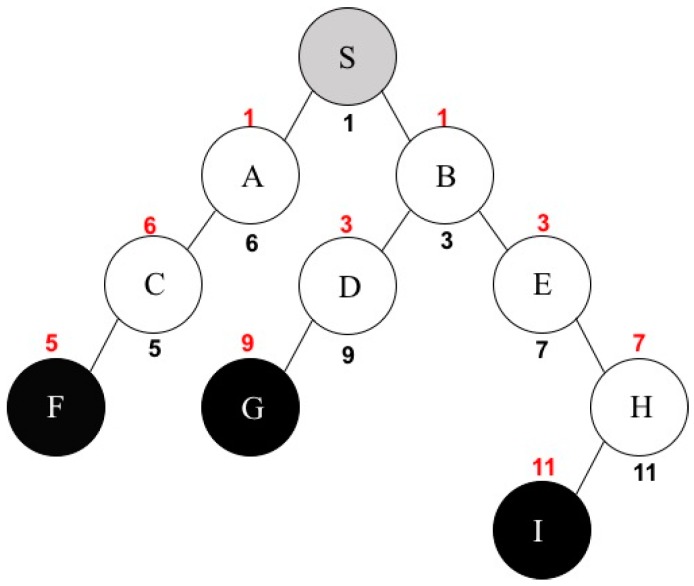
Channel assignment example by MICA.

The following scenario is a specific example in order to demonstrate the final step. Let us assume that *S_D_* = {A, H}, *S_N_* = {S, B, C, D, E}, nodes A and D have a channel separation of 3, and H and D have a channel separation of 2. One more assumption is that *CS <*A, D*>* is the maximum channel separation among all nodes in *S_N_*. Therefore, node D can have its own channel number based on this maximum value. Suppose that the channel number of *A* is 6 and the channel difference between A and D is 3, there might be one of the following conditions: the channel number of D is less than or equal to 3 (*COND*_1_) or that of D is greater than or equal to 9 (*COND*_2_). Therefore, D can have the channel number 3 or 9. In addition, assume that H uses the channel number of 11 and the channel separation between H and D is 2. The following two more conditions may be considered: the channel number of D is less than, or equal to, 9 (*COND*_3_) or that of D is greater than, or equal to, 13 (*COND*_4_). However, we cannot use 13 because our channel assumption is that we only have 11 channels from 1 to 11. Hence, a channel number of 13 is an invalid number to use, so *COND*_4_ will be eliminated. In order to satisfy three conditions from *COND*_1_ to *COND*_3_, MICA finally assigns a channel number of 9 to node D and puts it into *S_D_*. The reason why MICA cannot assign a channel number of 3 to D is that *COND*_2_ says the channel number of D should be greater than, or equal to, 9. This procedure continues until it covers all nodes in *S_N_*. Consequently, all nodes except F, G, and I, in a multicast tree are located in *S_D_*, which means that every node, except leaf nodes, has its own channel number of its sending interface. Figure 3 represents the multicast tree where every node has its own channel number for its sending and receiving interfaces. Leaf nodes F, G, and I have only a receiving interface because they do not need to forward packets and the source node S is simply a sending interface. In Figure 3, the black-colored number means the channel number for each node’s sending interface and the red-colored number represents the channel number for each node’s receiving interface, like Figure 1.

## 4. Simulations

We evaluate MICA by comparing it with the MCM algorithm. The reason why MICA is proposed is that it eliminates the drawback of MCM (HCP) by examining every pair of nodes in the network. Our simulation tool is QualNet 4.5 (SCALABLE Network Technologies, Culver City, CA, USA) [30]. For the first experiment, we measure an average number of packets received by multi-receivers and the standard deviation of average packets in different network topologies. In the experimental setting, the number of multi-receivers is fixed and a variety of multicast trees are used. For the second test, QualNet computes an average number of packets received by multi-receivers and an end-to-end packet delay with the different number of multi-receivers. In the second simulation setup, we use the same network topology during the entire simulation. Whenever carrying out the second experiment, only the number of multi-receivers is changed.

### 4.1. Performance Metrics

For a simulation study, the following metrics are used in order to measure the performance of MICA and MCM:

- *Average packet*: an average packet is defined as the average number of packets that each multi-receiver receives successfully during the simulation time.
- *Average delay*: an average delay is the average time taken for a packet to be transmitted across a network from source to destination.
- *Standard deviation*: the standard deviation is the variability or dispersion of packets received by all multi-receivers.

### 4.2. Simulation Parameters

We first perform 10 experiments with different network topologies. Whenever conducting each experiment, QualNet randomly places 30 wireless nodes in a flat area of 900 m by 900 m. In this scenario, there is one source and 10 multi-receivers in a multicast tree where these nodes are randomly selected as being a receiver. For the other experiment, a different number of multi-receivers is applied under the same topology. For the entire simulation settings, each node has two network interfaces for sending and receiving data packets, so they use two channel numbers for their radio interfaces. A total of 11 channel numbers are adopted for channel assignment. The transmission range of each wireless node is 250 m and the transmission rate is 11 Mbit/s. The data packet size for all traffic is 512 bytes and the transmission rate of a source node is 100 packets/s. The traffic model is a multicast constant bit rate (MCBR) traffic generator to evaluate the multicast performance. The QualNet software provides the following multicast routing protocols: on-demand multicast routing protocol (ODMRP) [31], distance vector routing protocol (DVMRP) [32], multicast open shortest path first (MOSPF) [33], and multicast ad hoc on-demand distance vector (MAODV) [34]. We decided to use MOSPF because this protocol basically supports multiple network interfaces. Finally, we set a total simulation time to 300 s. The aforementioned simulation parameters are summarized in Table 2.

### 4.3. Simulation Results

**Figure 4:**
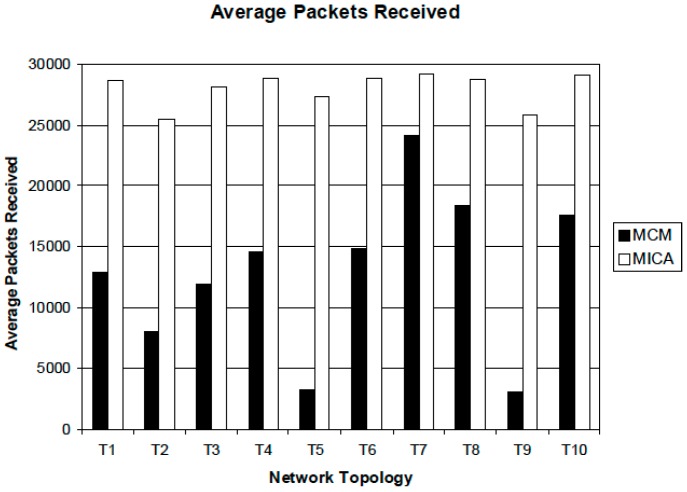
Average packets received by multi-receivers.

We generate different network topologies when performing the first scenario. Whenever executing each simulation, a source node produces 100 packets per second and the simulation time is 300 s, so a sender creates 30,000 packets during each simulation. For each experiment, we measure total packets received by each multi-receiver and compute the average number of received packets. These simulation results are represented in Figure 4. This graph shows the average number of packets received by multi-receivers during the simulation time. As seen from this graph, the performance of the proposed MICA algorithm is much better than that of MCM. The average number of packets received by multi-receivers using MICA is approximately between 25,000 and 29,000. This value is very close to the number of packets (30,000) the source generates. On the other hand, the majority of the average number of packets using MCM is below 20,000 and the worst case is under 5000.

**Figure 5:**
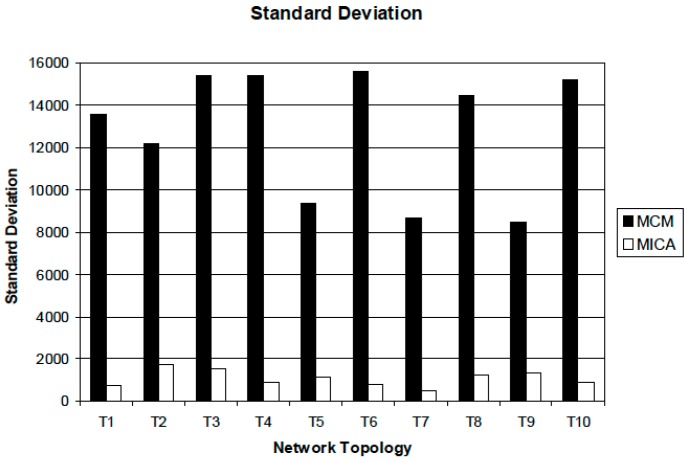
Standard deviation of all packets received by multi-receivers.

We also measured the standard deviation of all packets received by multi-receivers. The result is shown in Figure 5. This picture represents the standard deviation of the packets received by receivers. The standard deviation of MICA is much lower than that of MCM. For 10 experiments, the standard deviation of MICA was below 2000, which means all receivers receive packets evenly. However, MCM has a much higher value than MICA, which means some destinations receive some amount of packets but other nodes cannot receive a certain amount of packets. The standard deviation in our simulation shows the variability of packets received by all multi-receivers. If the value of standard deviation is small, all multi-receivers fairly receive data packets, but a large value depicts some receivers obtain enough packets, but others do not. Consequently, if the variance of packets received by multi-receivers is extremely large, then it indicates that overall network throughput is not outstanding.

**Figure 6:**
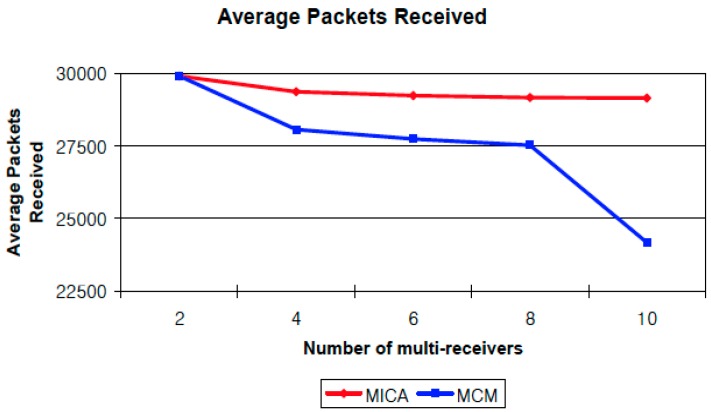
Impact of a different number of multi-receivers.

**Figure 7:**
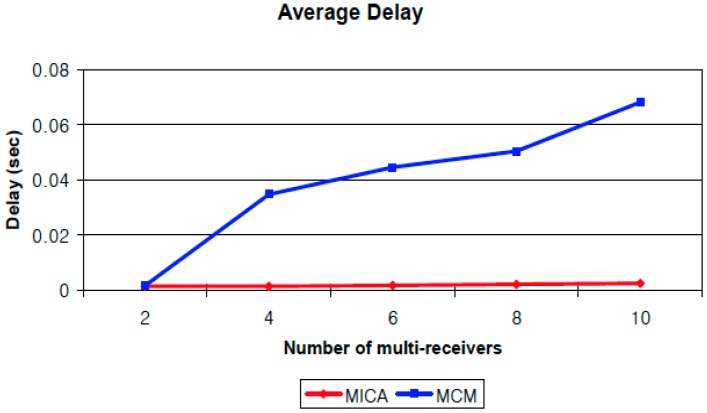
Comparison of end-to-end packet delay.

Finally, we calculate the average number of packets received in simulations with a different number of multi-receivers by assigning the number of receivers to 2, 4, 6, 8, and 10. The results are shown in Figure 6. This graph shows the trend of the average packets received as the number of receivers is increased. Although the number of receivers is increased, the performance of MICA is stable. All receivers receive almost all of the packets from the source regardless of the number of receivers. However, the performance of MCM is affected by the number of multi-receivers because the chance of network interference is also increased. We also compute the average end-to-end packet delay of MICA and MCM by comparing the average time for each packet takes to arrive at multi-receivers. Figure 7 shows the trend of average end-to-end packet delay. The average delay of MICA is almost same regardless of the number of receivers. However, the delay of MCM gets higher as the number of receivers is increases because of network interference. If network interference exists then it causes packet collision and retransmission, so the end-to-end delay is also increased.

The most important reason why we get these simulation results is that the MCM algorithm has HCP, which we discussed in Section 2. We conclude that HCP yields network interference among wireless nodes. As a result, it causes a poor network throughput. By eliminating this problem, MICA significantly improves the network performance for multicasting.

## 5. Conclusions

In this study, we propose a channel assignment algorithm for multicast in multi-channel multi-radio wireless mesh networks. We investigate the drawback of the MCM algorithm and try to find the solution to minimize network interference and enhance network throughput. MCM only considers one-hop neighbors for channel assignment. This mechanism may yield HCP, so there is network interference among wireless nodes when they communicate with each other at the same time and this problem might have an influence on overall network throughput. Our simulation results show that the performance of MCM is much worse than that of MICA because of network interference. Accordingly, our approach focuses on reducing network interference by considering every pair of nodes in a multicast tree for channel assignment. By minimizing interference among wireless nodes in a wireless network, we can improve overall network throughput and reduce end-to-end packet delay. The performance evaluation shows that our algorithm outperforms the MCM algorithm in terms of average packets received by multi-receivers and an average end-to-end delay in a given network environment.

## Figures and Tables

**Table 1:** Interference factor in an IEEE 802.11b network.

Channel Separation	2 Mbits/s	5.5 Mbits/s	11 Mbits/s
0	2.5	2.2	2.0
1	1.6	1.5	1.2
2	1.2	1.0	0.7
3	0.9	0.8	0.5
4	0.5	0.3	0.2
≥5	0.0	0.0	0.0

**Table 2:** Simulation parameters.

Parameter	Value
Number of channels used	11
Network size	30 nodes over 900 m × 900 m
Transmission range	250 m
Transmission rate at a physical layer	11 Mbits/s
Physical layer protocol	IEEE PHY 802.11b
Multicast routing protocol	MOSPF
Packet size	512 bytes
Transmission rate at an application layer	100 packets/s
Traffic model	MCBR
Simulation time	300 s
